# To screen or not to screen: an interactive framework for comparing costs of mass malaria treatment interventions

**DOI:** 10.1186/s12916-020-01609-7

**Published:** 2020-06-19

**Authors:** Justin Millar, Kok Ben Toh, Denis Valle

**Affiliations:** 1 School of Forest Resources and Conservation, University of Florida, Gainesville, USA; 2 Emerging Pathogens Institute, University of Florida, Gainesville, USA; 3 School of Natural Resources and Environment, University of Florida, Gainesville, USA

**Keywords:** Malaria, Decision support, Resource allocation, Data-driven decision-making, Mass drug administration, Mass screen and treat

## Abstract

**Background:**

Mass drug administration and mass-screen-and-treat interventions have been used to interrupt malaria transmission and reduce burden in sub-Saharan Africa. Determining which strategy will reduce costs is an important challenge for implementers; however, model-based simulations and field studies have yet to develop consensus guidelines. Moreover, there is often no way for decision-makers to directly interact with these data and/or models, incorporate local knowledge and expertise, and re-fit parameters to guide their specific goals.

**Methods:**

We propose a general framework for comparing costs associated with mass drug administrations and mass screen and treat based on the possible outcomes of each intervention and the costs associated with each outcome. We then used publicly available data from six countries in western Africa to develop spatial-explicit probabilistic models to estimate intervention costs based on baseline malaria prevalence, diagnostic performance, and sociodemographic factors (age and urbanicity). In addition to comparing specific scenarios, we also develop interactive web applications which allow managers to select data sources and model parameters, and directly input their own cost values.

**Results:**

The regional-level models revealed substantial spatial heterogeneity in malaria prevalence and diagnostic test sensitivity and specificity, indicating that a “one-size-fits-all” approach is unlikely to maximize resource allocation. For instance, urban communities in Burkina Faso typically had lower prevalence rates compared to rural communities (0.151 versus 0.383, respectively) as well as lower diagnostic sensitivity (0.699 versus 0.862, respectively); however, there was still substantial regional variation. Adjusting the cost associated with false negative diagnostic results to included additional costs, such as delayed treated and potential lost wages, undermined the overall costs associated with MSAT.

**Conclusions:**

The observed spatial variability and dependence on specified cost values support not only the need for location-specific intervention approaches but also the need to move beyond standard modeling approaches and towards interactive tools which allow implementers to engage directly with data and models. We believe that the framework demonstrated in this article will help connect modeling efforts and stakeholders in order to promote data-driven decision-making for the effective management of malaria, as well as other diseases.

## Background

Malaria continues to be a significant contributor to the global burden of diseases. Mass administration of antimalarial drug treatments (MDA) to entire populations has been used as an intervention strategy for reducing the global malaria burden [1, 2], particularly during elimination efforts in the early to mid-twentieth century [3]. Recently, however, interest in MDA as a viable malaria intervention strategy has reemerged, in particular in conjugation with emergency responses to non-malarial epidemics (e.g., the 2014–2015 Ebola outbreak in West Africa) [4–7] and seasonal malaria chemoprevention in the Sahel region [8].

Contemporary MDA interventions, primarily through intermittent preventive treatment and seasonal chemoprevention campaigns, have been used to interrupt malaria transmission in low endemicity settings [9], as well as reduce malaria burden in vulnerable subpopulations, such as young children and pregnant women, in high endemicity settings [10, 11]. In traditional MDA interventions, all individuals in a population or subpopulation receive treatment regardless of symptoms or other diagnostic information. This approach ensures that all sick individuals receive treatment; however, it also leads to overtreatment, which may increase the overall cost of MDA and undermine resource allocation. Modern antimalarial drugs, such as artemisinin-based combination therapy (ACT), can be expensive and often are in limited supply; therefore, wasting these resources on malaria-negative individuals can be costly [12, 13]. In addition, the overuse of ACTs can lead to an increase risk of anti-malaria resistance [14].

An alternative approach to MDA is mass screen and treat (MSAT), which consists of first screening the population with a diagnostic test and then only treating individuals with a positive test outcome. Because microscopic evaluations and molecular techniques (e.g., polymerase chain reaction) are often not a viable option in remote regions and/or at large operational scales, diagnosis is increasingly based on rapid diagnostics tests (RDTs) throughout much of sub-Saharan Africa [1, 15]. RDT screening has been repeatedly shown to be a viable, cost-effective option for diagnosing malaria [16, 17]. The widespread use of RDTs has significantly reduced the use of antimalarial drugs, helping to reduce the risk of resistance emergence [18, 19]. In addition to use of RDT in clinical settings, MSAT relying on RDT outcomes has been shown to be a cost-effective method for reducing malaria burden in certain contexts [20]. However, determining when and where MSAT will reduce the cost of traditional MDA is an important challenge [9]. On the one hand, despite the potential of MSAT to reduce costs and overtreatment, there have been notable failures in field studies in terms of ensuring long-term improvements in health and educational indices [21, 22]. The emergence of resistance is a particularly important concern given the growing consensus that repeated interventions are necessary for sustaining the impact of MDA and MSAT [9, 23–25]. A recent Cochrane review has indicated that 182 studies have assessed the impact of these types of malaria interventions (MDA and its variants, including MSAT) [2]; however, few guidelines have emerged to help decision-makers determine when MSAT is a more cost-effective strategy than MDA [26–29]. In general, MSAT is thought to be the preferred approach in high- to mid-transmission settings [30], which was supported by the study from Crowell et al. [3]. In contrast, however, Walker et al. [31] found that MDA was more cost-effective than MSAT in all but the highest transmission settings, and noted that the slight cost deficit in these areas was likely offset by the additional period prophylaxis provided to post-intervention infected individuals [32]. Gerardin et al. [33], on the other hand, argued that in control/pre-elimination settings, the cost of overtreatment by MDA may mitigate the detection advantage (i.e., ensuring all infected individuals are treated) and therefore undermine the cost-effectiveness of MDA compared to MSAT. Corroborating these findings with field research has been difficult, as much of the observed data on recent MDA and MSAT applications is held in gray literature and unpublished reports [2, 9].

Multiple factors can influence the costs of MSAT relative to MDA. For example, unlike MDA, inaccurate diagnostic results in MSAT can lead to both overtreatment and undertreatment. Although RDTs have high overall sensitivity (above 93%) and specificity (above 95%), a comprehensive review of field studies found substantial heterogeneity in RDT performance [34]. Additionally, the detection mechanism differs among different types of RDTs. For example, commonly used HRP2-based RDTs such as Paracheck® will fail to detect infections caused by non-*Plasmodium falciparum* species or by *P. falciparum* parasites which carry mutations to the HRP-2 gene, resulting in false negative results [35, 36]. False positive results can also be an issue, as the HPR-2 protein can persist in the host for up to 2 to 3 weeks after parasitemia has cleared [36]. Ultimately, the potential cost-savings advantage of MSAT over MDA depends on baseline likelihood of infection (i.e., prevalence), RDT sensitivity and specificity, the costs of treatment and RDTs, and the costs associated with false positive and false negative results [33, 37]. However, many of these factors can vary substantially within sub-Saharan countries [34, 37], and as a result, it can be difficult to generalize which strategy is likely to reduce implementation costs in each country/region [38]. Nevertheless, identifying the local optimal strategy is important for stakeholders and policy implementers as national malaria control programs move away from a “one-size-fits-all” approach.

In this article, we outline a conceptual framework for comparing the cost of malaria intervention strategies based on the probability of their possible outcomes and the costs associated with those outcomes, focusing on the comparison between MDA and MSAT. First, we demonstrate this comparative framework using hypothetical scenarios for each of these factors. Next, we create probabilistic models for estimating malaria prevalence and RDT performance using routinely collected national-scale survey data (e.g., Demographic Health Surveys (DHSs) and Malaria Indicator Surveys (MISs)) to present a real-world application. Finally, using these models, we build an interactive web application which allows end-users to compare the expected intervention costs in each region within each country based on the inputted economic values, thereby extending these models into decision support tools which allow implementers to interact with the data and models directly.

## Methods

### Estimating intervention costs

**Fig. 1:**
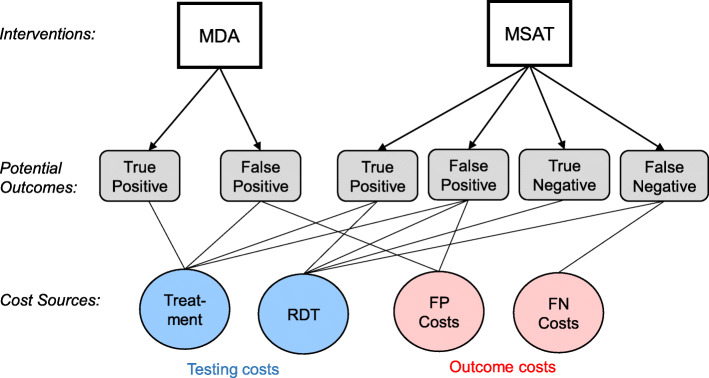
Conceptual framework for costs of mass drug administration (MDA) and mass screen and treat (MSAT). Flow diagram based on the potential outcomes and associated costs for each intervention. Testing and outcome costs are shown in blue and red, respectively. FP and FN stand for false positive and false negative, respectively

The expected cost per person associated with MDA and MSAT interventions can be estimated as a function of the costs of implementing these interventions, the costs associated with the potential outcomes, and the probability of those outcomes. The possible outcomes for an individual participant in an MDA campaign are either true positive or false positive, whereas an individual participant in an MSAT campaign may also be true negative or false negative (Fig. 1).

To determine the probability of each of these potential outcomes, we calculate the likelihood of malaria infection *p*(*M* = 1), the sensitivity *p*(RDT = 1 | *M* = 1), and specificity *p*(RDT = 0 | *M* = 0) of the screening diagnostic test (model variables are defined in Table 1). In this framework, testing costs refer to materials used by the intervention (e.g., RDT, treatment with an ACT), and outcome costs refer to additional costs related to outcomes from the intervention (e.g., additional healthcare costs due to a false negative RDT results). Cost items include cost of the diagnostic test used for screening (Cost_RDT_), the cost of antimalarial treatment (Cost_Trt_), and the outcome costs of false negatives (Cost_FN_) and false positives (Cost_FP_). Notice that the outcome costs of false negative or false positive diagnostic outcomes may incorporate multiple sources of cost (i.e., lost wages due to illness, deleterious impact of side effects, increased risk of antimalarial resistance) and that these costs may occur at varying levels of the overall healthcare system (e.g., provider costs, individual costs, societal costs). The per-person expected cost of MDA is given by:

$$ E\left[\mathrm{Cos}{\mathrm{t}}_{\mathrm{MDA}}\right]=\mathrm{Cos}{\mathrm{t}}_{\mathrm{Trt}}+\mathrm{Cos}{\mathrm{t}}_{\mathrm{FP}}\times p\left(M=0\right) $$

**Table 1:** Parameter definitions for expected cost equations

Equation parameters	Description
Cost components	
**Cost**_**Trt**_	Cost of treating one person (e.g., cost of one antimalarial drug)
**Cost**_**RDT**_	Cost of one rapid diagnostic test (RDT)
**Cost**_**FP**_	Cost associated with one false positive outcome
**Cost**_**FN**_	Cost associated with one false negative outcome
Outcome probabilities	
***p***(***M =*1**)***,p***(***M =*0**)	Likelihood of microscopy outcome (1 = infected, 0 = uninfected)
***p***(**RDT*=*0**)**,*p***(**RDT*=*1**)	Likelihood of RDT outcome (1 = positive, 0 = negative)
***p***(**RDT*=*1** | ***M =*0**)	Likelihood of false positive
***p***(**RDT*=*0** | ***M =*1**)	Likelihood of false negative

The per-person expected cost of MSAT is given by:

$$ E\left[\mathrm{Cos}{\mathrm{t}}_{\mathrm{MSAT}}\right]=\mathrm{Cos}{\mathrm{t}}_{\mathrm{RDT}}+\mathrm{Cos}{\mathrm{t}}_{\mathrm{Trt}}\times p\left(\mathrm{RDT}=1\right)+ $$

$$ \mathrm{Cos}{\mathrm{t}}_{\mathrm{FP}}\times p\left(\mathrm{RDT}=1|M=0\right)+\mathrm{Cos}{\mathrm{t}}_{\mathrm{FN}}\times p\left(\mathrm{RDT}=0|M=1\right) $$

where, based on the law of total probabilities, *p*(RDT = 1) is given by:

$$ p\left(\mathrm{RDT}=1\right)=p\left(M=1\right)\times p\left(\mathrm{RDT}=1|M=1\right)+p\left(M=0\right)\times p\left(\mathrm{RDT}=1|M=0\right) $$

This framework could be augmented through the inclusion of additional layers of complexity, such as the inclusion of overall program-level costs and/or an expanded set of possible outcomes (e.g., likelihood of developing severe malaria and the associated costs), if data on these costs and outcomes were available. We elected to develop this individual-level framework which includes productivity losses, but note that it can be readily extended to more complex data and applications such as designating societal and healthcare provider costs.

We used this framework to compare the potential cost of MDA and MSAT in two contexts. In the generalized comparisons, we compare costs across all possible baseline prevalence. The sensitivity, specificity, and costs of RDTs were based on a recent report from the WHO [26] and make comparisons using hypothetical scenarios based on differing costs associated with false negative and false positive outcomes (Table 2). In the context-specific comparisons, we use publically available data to construct models for estimate baseline prevalence as well as RDT sensitivity and specificity, and apply similar scenario-based comparisons.

**Table 2:** Diagnostic performance and costs (in USD) used for baseline comparison

*Diagnostic accuracy*		
RDT sensitivity^*^	0.82 to 0.96	
RDT specificity^*^	0.80 to 0.90	
*Healthcare provider costs (USD)*		
Cost of RDT^*^	0.60	
Cost of treatment (ACT)^*^	2.40	
*Societal costs (USD)*		
Lost daily wages^†^	21.55	
*Scenario costs*		
Scenario	False negatives	False positives
A. No cost associated with false positive or false negative	0.00	0.00
B. False negative equal to cost of RDT^*^	2.40	0.00
C. False negative equal to cost of RDT^*^ and potential lost wages^†^	23.95	0.00
D. Same as C with small offset for false positives	23.95	1.50

*Based on [26]

^†^Based on [39]

### Modeling outcome probability based on prevalence, sensitivity, and specificity

#### Description of the data used for modeling

Information on malaria status of children 5 years old and under was sourced from Demographic and Health and Malaria Indicator Surveys (DHS and MIS, respectively). These data are freely available, use standardized sampling procedures, and contain information on a broad range of malaria indicators, such as age, urbanicity, and fever history. Recent surveys were selected for Burkina Faso [40], Cote d’Ivoire [41], Ghana [42], Guinea [43], Nigeria [44], and Togo [45]. For each survey, the data are reported at the first order civil entity below the country level (commonly referred to as “administrative area 1”). The official names for these areas vary between countries; therefore, we adopt the term “regions” throughout the article for clarity. Note that this means that “regions” are operationally defined as sub-areas within individual countries. These West African countries were selected because they each contain relatively recent standardized country-wide surveys and included information on RDT and microscopy (assumed to be the “gold standard” in this region [46, 47]). The RDTs used in these are surveys are specific to *Plasmodium falciparum*, which account for the vast majority of malarial infections in this region. Individual survey sample sizes ranged from 2713 to 6112 individuals, distributed across 6 to 13 regions per country (Table 3).

**Table 3:** Summary of data sources

Country	Survey type	Collection period	Sample size	Regions
Burkina Faso	Malaria Indicator Survey [48]	September 2014–November 2014	6112	13
Cote d’Ivoire	Demographic Health Survey [49]	December 2011–May 2012	3344	11
Ghana	Demographic Health Survey [50]	September 2014–December 2014	2713	10
Guinea	Demographic Health Survey [51]	June 2012–October 2012	3198	11
Nigeria	Malaria Indicator Survey [52]	October 2015–November 2015	5127	6
Togo	Demographic Health Survey [53]	November 2013–April 2014	3215	6

#### Modeling prevalence, sensitivity, and specificity

Malaria prevalence and diagnostic performance were estimated separately for each country using Bayesian mixed-effect logistic regression models. Microscopy (*M*) was considered the “gold standard” for detecting malaria infections in this region [46, 47], and RDT (*R*) was considered the screening diagnostic test. Let *M*_*ijk*_ represent the binary infection status (as determined by microscopy) of individual *i* from cluster *j* in region *k*. We assume that *M*_*ijk*_ is given by:

$$ {M}_{ijk}\mid {p}_{ijk}\sim \mathrm{Bernouli}\left({p}_{ijk}\right) $$

where *p*_*ijk*_ is the probability of infection (e.g., prevalence). We constructed the model using just two basic covariates that could be relevant for the development of region-specific policy, namely age in months (Age_*ijk*_) and a binary classification of urban/rural environment (Urban_*jk*_) based on the survey’s definition (see references in Table 3). Using the logit link function $g(p)=\log \left(\frac{p}{1-p}\right)$, we model infection probability as:

$$ g\left({p}_{ijk}\right)={a}_{jk}+{\beta}_{0,k}+{\beta}_{1,k}\times \mathrm{Ag}{\mathrm{e}}_{ijk}+{\beta}_{2,k}\times {\mathrm{Urban}}_{jk}+{\beta}_3\times \mathrm{Ag}{\mathrm{e}}_{ijk}+{\beta}_4\times {\mathrm{Urban}}_{jk} $$

This equation includes a cluster-level random-effect intercept *a*_*jk*_, regional-level fixed-effects (i.e., intercepts *β*_0, *k*_ and slopes *β*_1, *k*_ and *β*_2, *k*_), and country-level fixed-effects (i.e., country-level slopes *β*_3_ and urbanicity *β*_4_).

In relation to RDT, let *R*_*ijk*_ represent the binary test outcome (1 for positive, 0 for negative) of individual *i* from cluster *j* in region *k*. We assume that:

$$ {R}_{ijk}\mid {M}_{ijk}=1,{Sn}_{ijk}\sim \mathrm{Bernouli}\left({Sn}_{ijk}\right) $$

$$ {R}_{ijk}\mid {M}_{ijk}=0,{Sp}_{ijk}\sim \mathrm{Bernouli}\left(1-{Sp}_{ijk}\right) $$

where *Sn*_*ijk*_ and *Sp*_*ijk*_ denote the RDT sensitivity and specificity, respectively. Both parameters were modeled with the same set of predictor variables and link function as *p*_*ijk*_.

Individual models for prevalence, sensitivity, and specificity were created for each country using the “brms” package in the open-source R statistical software [48, 49]. We adopted the recommended priors based on our model specification (flat priors for the fixed effects, and half Student’s *t* with 3 degrees of freedom and a scaling factor of 1 for the standard deviation of the random effects) [49–51]. Due to the large sample size in our data, these priors play very little to no role in influencing our results. The fully specified model with the full posterior is described in Supplemental Material 1. Each model was fitted using a Hamiltonian Monte Carlo with 4 independent chains, each containing a 1000-iteration burn-in phase and a 1000-iteration sampling phase, resulting in 4000 posterior samples. Parameter convergence was determined using the potential scale reduction factor (convergence at $\hat{R}<1.05$) [52].

### Designing the interactive framework

Based on the outlined cost functions and associated outcome probabilities, we developed an interactive framework which allows users to compare the relative costs of MDA and MSAT in each region (as defined by DHS/MIS) within each country. This was done using the “shiny” package in R [53], a package that enables the creation of web-based interactive applications directly from R code (instead of HTML, CSS, or JavaScript), which can then be freely hosted and accessed on the Internet. Web applications like this can facilitate engagement with stakeholders and policymakers with limited statistical and programming backgrounds. Examples of other epidemiological interactive tools developed in Shiny can be found in [54–57].

By using probabilistic models for specifying the outcome probabilities, we are able to compare intervention scenarios while incorporating uncertainty. Aside from inputting cost values, the interface allows users to select covariate values (i.e., country, age range, and urbanicity), which then results in an update of the cost comparison in real time. We used the “leaflet” package in R to create an interactive map-based visualization of the cost comparison [58]. The code used to create this tool is available at https://github.com/justinmillar/mda-msat. We also constructed a “generalized” version of the application, which allows the user to specify a range for RDT sensitivity and specificity and compare MDA versus MSAT over all possible prevalence rates (rather than estimating these parameters from data).

## Results

### General comparisons

**Fig. 2:**
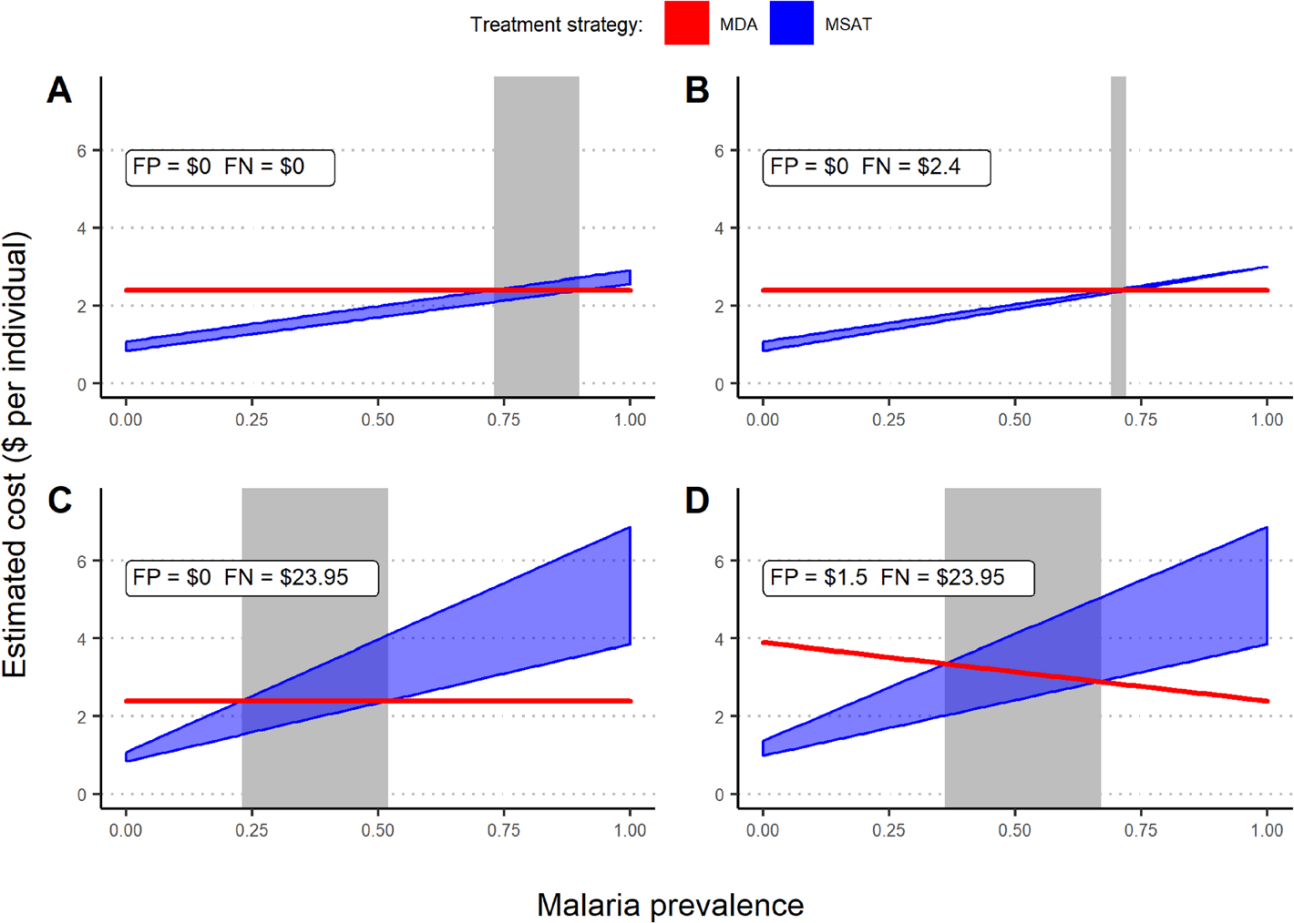
Costs of mass drug administration (MDA) and mass screen and treat (MSAT) based on malaria prevalence. Each panel depicts a different scenario relative to the costs associated with false positive (FP) and false negative (FN) outcomes, as specified in the legends. The lower estimated cost (*y*-axis) indicates which strategy will have lower associated costs for a given prevalence rate (*x*-axis). RDT sensitivity and specificity ranged from 0.82 to 0.96 and 0.80 to 0.90, respectively, and the cost of treatment and RDT were set to $2.40 and $0.60, based on a WHO report [26]. The gray-shaded region indicates overlap in expected cost, where the more favorable strategy is unclear due to the range of possible values for RDT sensitivity and specificity

Using general cost and diagnostic accuracy parameters from the WHO [26] (Table 2), MSAT is preferred in nearly all but the highest disease burden settings when the cost of false negatives is ignored (Fig. 2a). However, the costs associated with MSAT increase in higher prevalence scenarios once the cost of false negatives is assumed to be equal to the cost of treatment (e.g., an RDT-negative individual eventually receives treatment; cost of $2.40 [26]) (Fig. 2b). The individual-level cost of MSAT can be further undermined if the overall economic burden of false negative includes additional costs. To demonstrate this, if we assume a scenario where a person whose child has a false negative result also incurs a day of lost wages (cost of $23.95, based on hourly rate and assuming 8 h per workday based on the median monthly income in sub-Saharan from World Bank estimates [39]), in order to take their child to a health facility to receive care, then screen and treat yields a significantly higher costs (Fig. 2c). Note that this is just one potential scenario where the cost of a false outcome can drastically shift the associated costs. Other scenarios, such as a false negative leading to a severe malaria infection, could decrease the benefit of MSAT. Finally, the primary effect of including a cost associated with false positive is raising the cost of MDA treatment in lower burden settings, which is eventually offset by costs associated with misdiagnosis in the screen-and-treat scenario as prevalence increases (Fig. 2d).

In the following section, we present similar scenario-based realizations using the models fit with national survey data. These scenarios represent just a small subset of all the possible scenarios. We believe this demonstrates the utility of using interactive decision support tools, which can re-create cost comparisons based on user-defined scenarios. Also note that these models are fit using data from young children (6 to 59 months old), and therefore, the displayed results are related to this particular subpopulation.

### Context-specific observations based on national-scale survey data

Individual models for malaria prevalence and RDT sensitivity and specificity rates for young children were fitted for all six country datasets. In each model, all parameters reached convergence based on the potential scale reduction factor (all $\hat{R}$ values ranged between 0.999 and 1.012). Details on each model are provided in Supplemental Material 1, and the regional prevalence, sensitivity, and specificity estimates are provided in Supplemental Material 2. The following sections illustrate the influence of the regression parameter estimates, cost scenarios, and parameter uncertainty on the cost comparison using survey data from Burkina Faso as a representative example.

#### Effect of false negatives

**Fig. 3:**
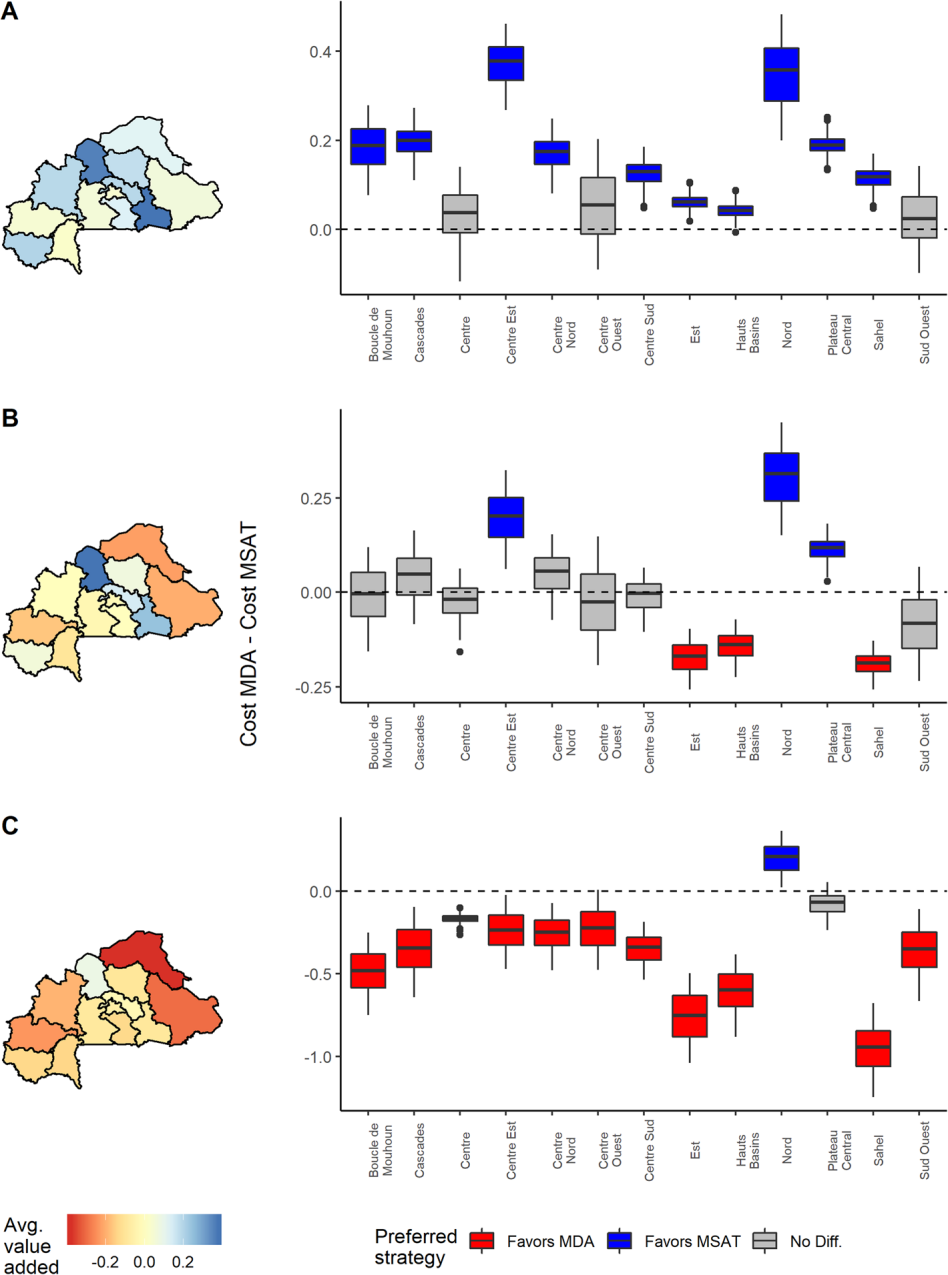
Value added from screen then treat among rural communities in Burkina Faso. Regional maps of the mean value added (i.e., MDA costs minus MSAT costs) and boxplots of value added estimates are shown on the left and right panels, respectively. Positive values (blue) indicate regions where MSAT is favored whereas negative values (red) indicate regions where MDA is favored. Whiskers in boxplots indicate 95% credible intervals. When these intervals contain both positive and negative values, there is no significant difference regarding costs between strategies (gray). Cost of diagnostic test (RDT) and treatment were set at $0.60 and $2.55, respectively. **a** Added value estimates ignoring any potential costs associated with false negative results. **b** Value added estimates when cost associated with false negative results is set to the cost of receiving delayed treatment. **c** Value added estimates when cost associated with false negative results includes the cost of receiving delayed treatment and 1 day of lost wage (based on minimum wage [39]). In all of these scenarios, we assume no cost associated with false positive outcomes

Designating costs specifically related to incorrect diagnostic results can have profound impacts on the cost of the MSAT. Consider the rural communities in Burkina Faso (Fig. 3), which fall within the mid- to high-transmission setting where MSAT is considered to be viable and potentially cost-effective relative to MDA, and where both clinical trials and mathematical studies have examined the effectiveness of MSAT [22, 24, 59]. We chose a favorable cost setting for MSAT by setting RDT cost to $0.60 and antimalarial treatment cost to $2.55. These costs correspond to the lower and higher ends of RDT and antimalarial prices, respectively, based on a recent WHO report [26]. Figure 3a shows the estimated value added from screening per individual for rural communities in Burkina Faso assuming no cost associated with false negatives or false positives. As expected, we find that under these conditions, MSAT is favored in most regions in Burkina Faso, and there are no regions that favor MDA. However, MSAT becomes relatively more costly once we attribute cost to false negative outcomes. When the cost of false negatives is set to the cost of the antimalarial treatment, which corresponds to a scenario where all truly infected individuals will eventually pay to receive treatment, MSAT becomes less favorable. MSAT is only favored in three regions (which had relatively low prevalence and higher sensitivity), and there are more regions where MDA may be more favorable or there is little expected cost difference between MDA and MSAT (Fig. 3b). MSAT becomes relatively more costly as the cost associated with false negatives increases. Under the hypothetical scenario where a false negative also incurs a lost 1 day’s wage based on the minimum wage in Burkina Faso ($6.37 per day, based on [39]), MDA is favored in all but two regions, despite relatively high prevalence rates (ranging from 0.26 (CI 0.18–0.36) to 0.66 (CI 0.53–0.77)) (Fig. 3c).

#### Differences between urban and rural communities

**Fig. 4:**
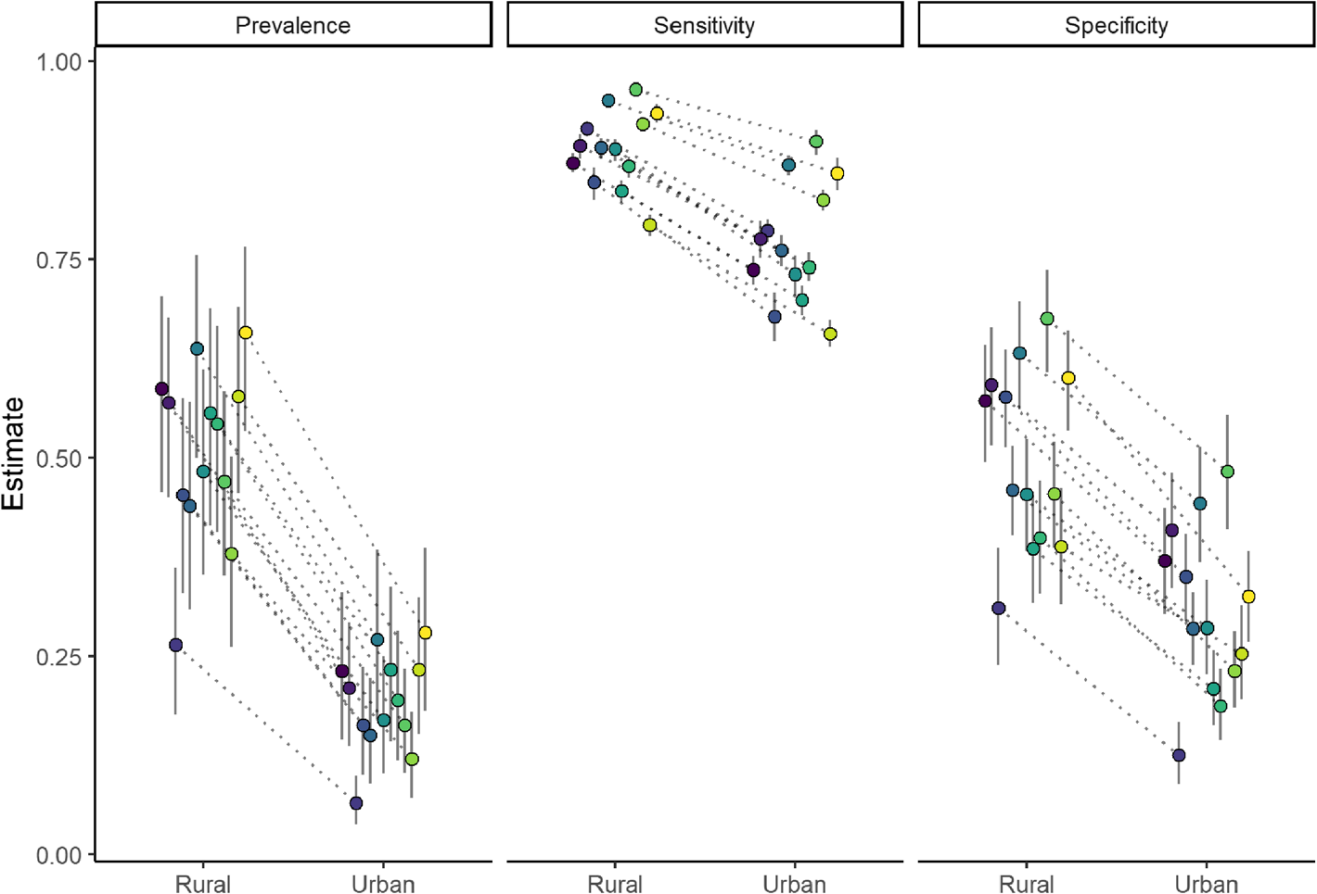
Comparison of regional-level differences of malaria prevalence and RDT sensitivity and specificity in Burkina Faso for urban and rural areas. Mean value estimate (circles) and 95% credible interval (vertical bars) for each region are based on 1000 posterior draws from each model. Each region has a unique shade and is connected by a dotted line to depict differences in parameter estimates between urban and rural communities

**Fig. 5:**
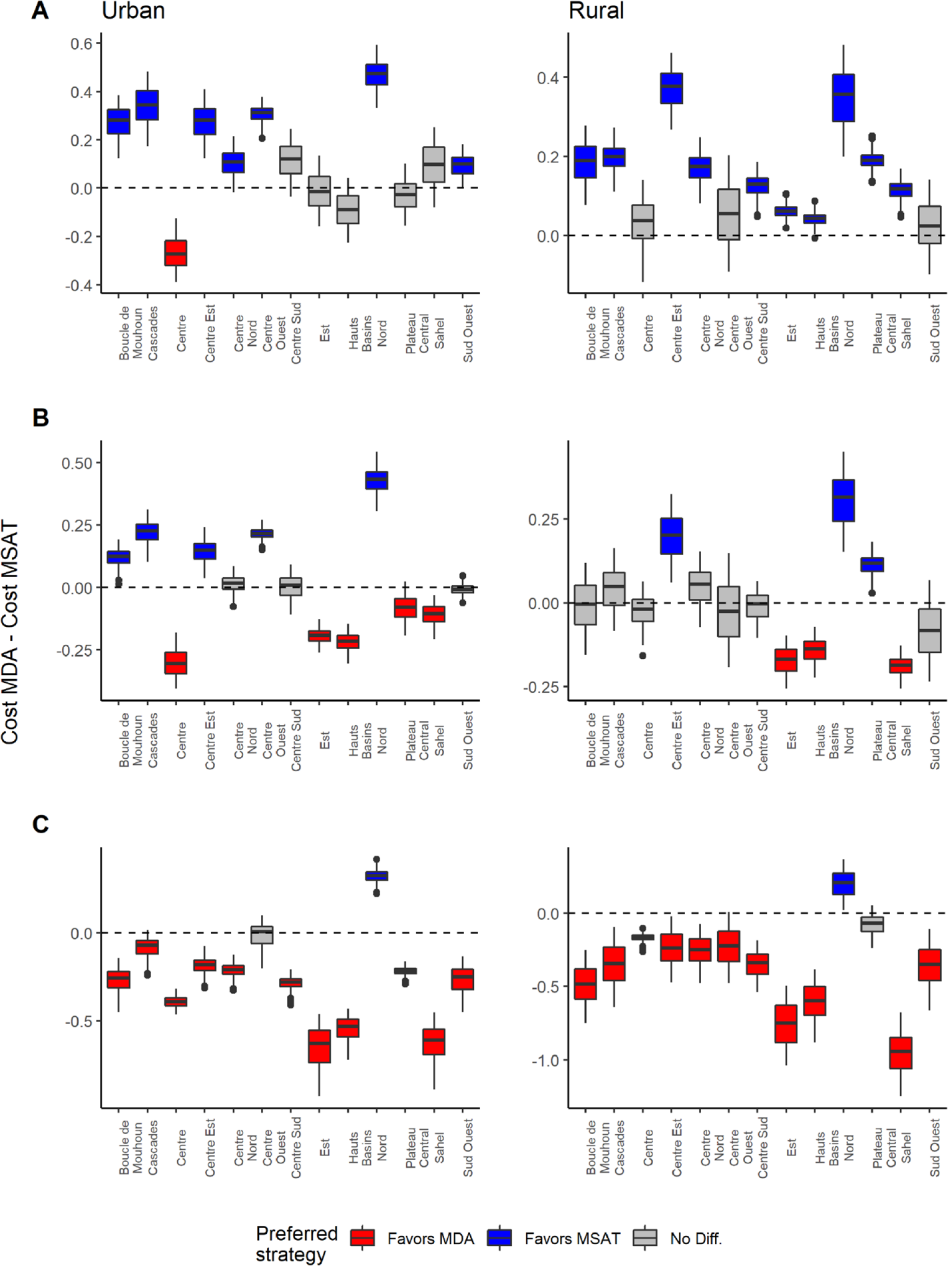
Urban versus rural comparison of value added from screen then treat in Burkina Faso. Comparison of regional value added (per individual) from diagnostic screening (MDA costs minus MSAT costs) between urban (left plots) and rural (right plots) communities. Positive values (blue) favor MSAT, negative values (red) favor MDA, and 95% interval ranges that contain both positive and negative values indicate no significant difference (gray). Cost of diagnostic test (RDT) and treatment were set at $0.60 and $2.55, respectively. **a** Added value estimates ignoring any potential costs associated with false negative results. **b** Value added estimates when cost associated with false negative results is set to the cost of receiving delayed treatment. **c** Value added estimates when cost associated with false negative results includes the cost of receiving delayed treatment and 1 day of lost wage (based on minimum wage [39]). In all of these scenarios, we assume no cost associated with false positive outcomes

As outlined earlier, there are many factors which influence malaria prevalence and RDT performance, and therefore may influence the costs of MDA and MSAT. One factor that strongly determines these variables is the differences between urban and rural communities. Although there is considerable regional-level variability in malaria prevalence within rural communities in Burkina Faso, rural communities consistently have higher prevalence than their urban counterparts (Fig. 4). Regional RDT sensitivity and specificity rates also consistently decline from rural to urban communities (see [34] for RDT performance on varying baseline prevalence). These differences between urban and rural communities can affect the costs of both intervention strategies. Figure 5 depicts the same cost scenarios as Fig. 3 for both rural and urban communities in Burkina Faso. These comparisons generally indicate that higher prevalence rural communities will tend to favor MSAT while lower prevalence urban communities tend to favor MDA, although these comparisons greatly depend on cost assumptions. The Nord region was the only region where both urban and rural communities favored MSAT in all cost scenarios. Interestingly, this was not linked to the regional prevalence rates, which were not too different from the other regions (0.16 and 0.47 for urban and rural communities, respectively), but instead were associated with having the highest sensitivity and specificity rates for both community types. This result suggests that even under a cost scenario which strongly favors one approach (in this case MDA), there was still a region where MSAT was favored, which highlights the importance of diagnostic accuracy in assessing costs at the country level.

#### Estimating regional breakpoints for treatment and RDT costs

**Fig. 6:**
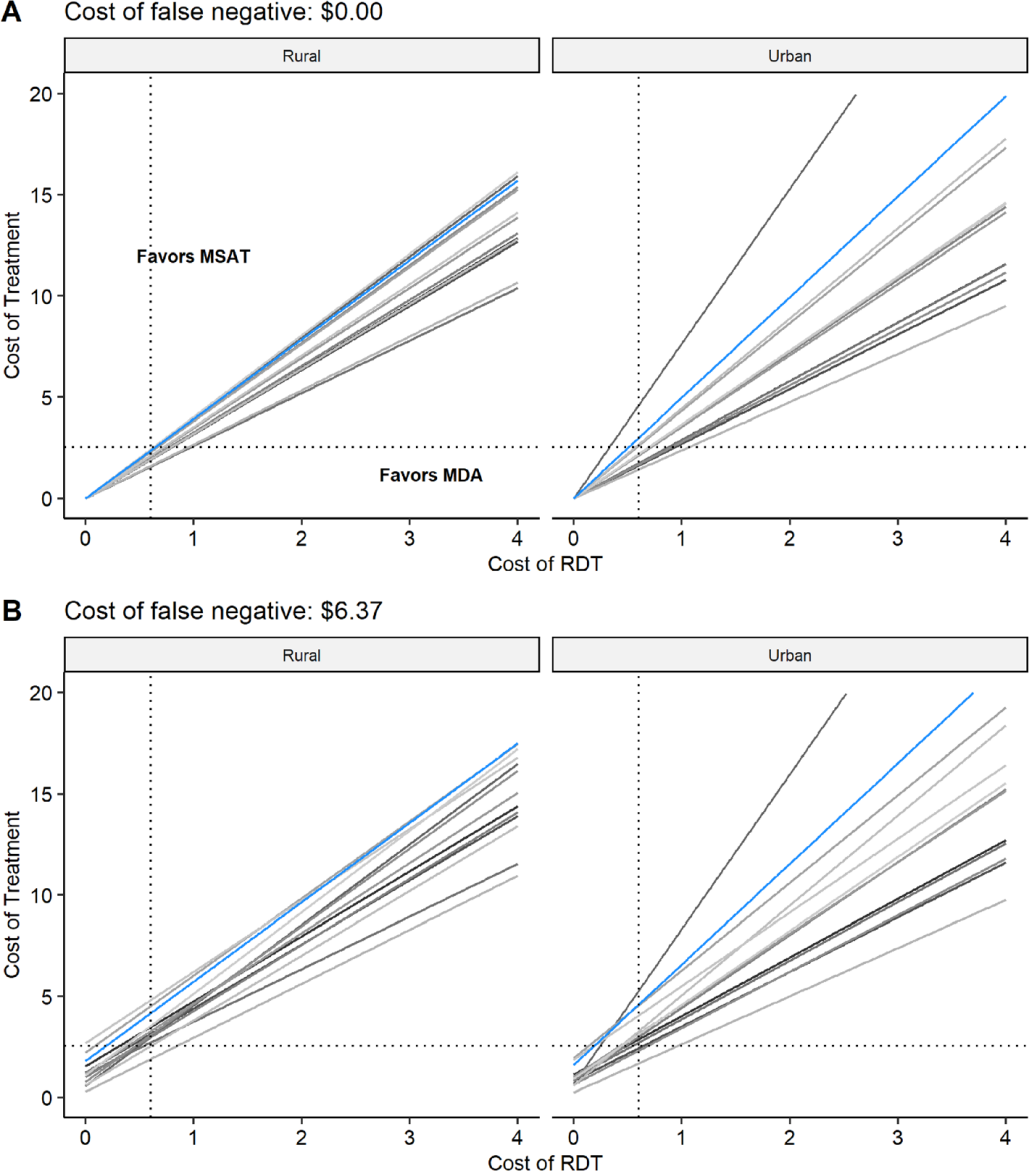
Regional breakpoints for cost of treatment and diagnostic test (RDT) in Burkina Faso. Cost points above the line favor MSAT in that region, whereas cost points below the line favor MDA. Rural and urban communities are depicted on the left and right columns, respectively. **a** No cost associated with false negative results. **b** A cost associated with false negative results which includes the cost of 1 day of lost wages (based on minimum wage [39]). Each line represents a different region, and the blue line emphasizes the Hauts-Bassins region. In all of these scenarios, we assume no cost associated with false positive outcomes

In addition to comparing the costs of MDA and MSAT directly based on specific values for the direct costs of treatments and diagnostic tests, it may also be valuable to identify the breakpoints for these costs. For example, under different scenarios of false positive and false negative costs, we can use the estimated regional malaria prevalence, RDT sensitivity, and RDT specificity to determine the cost of RDT and treatment for which MDA (or MSAT) would reduce costs. Figure 6 illustrates this using the scenarios in the previous examples for Burkina Faso, assuming no cost associated with false negatives (Fig. 6a) and including loss of 1 day’s wage in the cost associated with false negatives (Fig. 6b). Notice that as expected, for a given cost of RDT, the cost comparison will tend to favor MSAT as the price of treatment increases. Similarly, for a given cost of treatment, MDA is favored as RDT cost increases. This breakpoint tends to be lower in rural communities, which typically have higher prevalence rates, and increases when we include costs associated with false negative results. For example, the dashed vertical and horizontal lines demonstrate this relationship using the RDT and treatment cost for the previous example ($0.60 and $2.55, respectively). Based on these costs, MDA will be favored in most rural districts whereas MSAT may be favored in the urban communities for some districts. These figures illustrate the critical role of the ratio of treatment cost and screening cost in comparing the costs of these interventions and that regional characteristics (prevalence and diagnostic test performance) strongly mediate the relationship between this cost ratio and differences in intervention costs. For example, the Hauts-Bassins region is highlighted to demonstrate how including an outcome cost associated with false negative results in a substantial shift towards favoring MSAT.

### Interactive applications

The application based on the “General comparison” section is available at https://jjmillar.shinyapps.io/msat-general/. Following the conceptual framework (Fig. 1), this tool allows the user to set the costs of treatment and diagnostic test (RDT), the costs of false positive and false negative outcomes, and a range for the potential diagnostic sensitivity and specificity. The tool relies on these user-defined inputs to estimate the cost of MDA and MSAT across all possible prevalence, generating an output similar to the plots in Fig. 3. The application based on the data from national surveys and modeling outcomes from prevalence, sensitivity, and specificity is available at https://jjmillar.shinyapps.io/msat-example/. This tool allows the user to select the country and setting (e.g., age range and urban or rural communities), as well as the same cost parameters in the first tool. The outputs are similar to the comparisons shown in Figs. 4, 5, and 6.

## Discussion

As described in the comprehensive review by Newby et al. [9], many studies have been published on the impact and effectiveness of MDA and MSAT. Implementation of both interventions has experienced both successes and failures. Moreover, simulations based on mathematical models comparing the costs of MDA and MSAT have in some cases provided conflicting recommendations [3, 31–33]. Rather than using mathematical models, our contribution to this important question of resource allocation focuses on using statistical models to quantify the likelihood of different outcomes associated with each intervention. In addition, we use these statistical models to power interactive decision support tools, in which users set the cost parameters and interact directly with inference from our statistical models in an open-source, cross-platform format.

We have prioritized a methodology that would be maximally flexible and accessible, including using familiar publicly available data and relying on relatively simple statistical models and tools developed in open-source and free software. There are, however, several notable limitations associated with these data. First, in order to model malaria status and diagnostic sensitivity and specificity, we assumed microscopy to be the gold standard. While microscopy is considered to be the gold standard in western sub-Saharan Africa [46, 47], it has been well established that microscopy-based diagnoses are imperfect [47, 60, 61] and that several factors (e.g., parasite density) can influence the accuracy of microscopy [62]. Additionally, while microscopy prevalence and RDT prevalence are strongly correlated, other research on DHS data has shown that this relationship is not always linear and that there are additional factors (such as the proportion of febrile individuals) that can influence this relationship [63]. By using microscopy as a gold standard, our analysis also ignores submicroscopic infections, which typically improve the cost-effectiveness of MDA relative to MSAT. Submicroscopic infections can be significant reservoirs for sustaining transmission; however, they are most impactful in older populations in low-transmission settings and elimination-phase interventions [24, 64, 65], rather than young children in endemic settings. We believe that data on more precise diagnostic tests (such as polymerase chain reaction) should be used if they were available at national scales. Our methodology can easily be modified to produce better estimates of RDT sensitivity and specificity that accounts for submicroscopic infection, leading to improved cost comparisons.

Second, the approach for estimating costs of MDA and MSAT presented in this article is intentionally simplistic because data that would support a more comprehensive national-level analysis for multiple West African countries were not available. For instance, in addition to the individual-level costs, there are also fixed programmatic-level costs of implementing each intervention, which are unlikely to be equivalent for MDA and MSAT (e.g., RDT-based intervention required additional training and storage). Furthermore, we discuss the individual-level cost of receiving the intervention, but do not take into account how these interventions influence morbidity/mortality metrics (e.g., disability-adjusted life-year). Including data on incremental outcomes would allow for comparing effectiveness, rather than just overall costs. In particular, determining which metric to use for the cost-effectiveness comparison is important for defining the goal of the intervention (e.g., interrupting transmission or reducing malaria burden). We also note that the simplified cost analysis relying on productive losses may disproportionally favor MSAT in some of the hypothetical scenarios. More detailed cost analyses, including stratified cost sources, are still needed and may yield different results to the hypothetical scenarios used to demonstrate the framework. These could be implemented by designing additional compartments to the cost components in Table 1.

Finally, if national-level data on malaria prevalence and RDT performance were available for febrile patients seeking help at health facilities, tools like the one described in this article could be developed for clinical settings to determine where and when test and treat would be a better option than presumptive treatment. In this case, one could also account for other potential outcomes, such as developing severe malaria (which may have a much higher associated cost), not adhering to a diagnostic test result, and the development of adverse side effects to treatment. Such a tool may be useful for improving adherence to national policies regarding treatment protocols [66–68].

The WHO recently identified a pressing need for modeling-based approaches to guide the selection of optimal interventions under different epidemiological conditions [69]. Decision support tools designed specifically for malaria control, particularly using mapping approaches and geostatistical models, have become more prevalent in recent years as national survey data have become more broadly accessible [70–73]. Our framework aims to provide a decision support tool for stakeholders for comparing costs associated with MDA or MSAT in different regions. We have demonstrated that such tools can be created and adapted using a standard, open-source program, helping to bridge the gap between methodological advancements and real-world decision-making. This is an important extension, as traditional scientific articles are often not an effective way to communicate the practical implications of complex analysis to policymakers [74]. These decision support tools are critically important as national malaria control programs have identified the need to move away from “one-size-fits-all” intervention and require tools for identifying optimal interventions based on location-specific conditions [75]. The applications presented in this article contribute to the growing pool of decision support tools for guiding malaria control interventions.

Similar to the work by Lubell et al. [70], one valuable characteristic of the support tool presented is interactivity. Standard results presented in scientific articles are limited to the parameters or scenarios selected by scientists, which often do not necessarily match those that would have been chosen by decision-makers. Furthermore, often, decision-makers must consider additional information that is either unknown or unaccounted for by the original developer, which is only possible if users can explore different scenarios within the decision support tool. However, similar to using statistical models without understanding their underlying assumptions, decision support tools can be prone to misinterpretation if not carefully designed and described [76]. Furthermore, important properties of the tools presented in this article are that they rely solely on a freely available open-source program, can be hosted on web applications (which makes them platform-independent), and can be completely built using software regularly used in epidemiological research. Finally, by using a Bayesian framework for modeling the data and enabling user-defined inputs for cost parameters, our application allows stakeholders to make informed decisions while taking into account uncertainty in the outcomes under different cost scenarios.

## Conclusion

We present a flexible framework for comparing the cost of MDA and MSAT and use this framework along with publicly available malaria data from national-scale surveys to construct an interactive decision support tool. The methodology used to create this tool addresses critical issues (e.g., cross-platform, open-source, real-time interactivity) with previous decision support tools for guiding malaria interventions and can be built using widely used open-source software. The tool provides a platform for decision-makers (who may not have a strong statistical background) to interact with statistical models and adjust the parameters to fit their context and external knowledge in order to support data-driven decision-making. We believe that similar decision support tools designed to fit specific malaria interventions and contexts will be valuable assets for guiding data-driven decision-making for malaria control and elimination in a way that recognizes the inherent differences between regions.

## Supplementary information

**Additional file 1.**

**Additional file 2.**

## Data Availability

The codes used to create this analysis, including the modeling outcomes and interactive applications, are available at https://github.com/justinmillar/mda-msat. The Shiny applications are available at https://jjmillar.shinyapps.io/msat-example/ and https://jjmillar.shinyapps.io/msat-general/. The GitHub repository also details how to run the applications locally in R. The data were sourced from publicly available national surveys, found in Table 3.

## Abbreviations

ACT: Artemisinin-based combination therapy
DHS: Demographic and Health Survey
MDA: Mass drug administration
MIS: Malaria Indicator Survey
MSAT: Mass screen and treat
RDT: Rapid diagnostic test
WHO: World Health Organization

## References

[1] Greenwood B (2004). The use of anti-malarial drugs to prevent malaria in the population of malaria-endemic areas. Am J Trop Med Hyg.

[2] Poirot E, Skarbinski J, Sinclair D, Kachur SP, Slutsker L, Hwang J. Mass drug administration for malaria. In: Cochrane Database of Systematic Reviews [Internet]: Wiley; 2013. Available from: http://onlinelibrary.wiley.com/doi/10.1002/14651858.CD008846.pub2/abstract. 10.1002/14651858.CD008846.pub2 PMC4468927 24318836

[3] Crowell V, Briët OJ, Hardy D, Chitnis N, Maire N, Pasquale AD (2013). Modelling the cost-effectiveness of mass screening and treatment for reducing Plasmodium falciparum malaria burden. Malar J.

[4] Aregawi M, Smith SJ, Sillah-Kanu M, Seppeh J, Kamara ARY, Williams RO (2016). Impact of the mass drug administration for malaria in response to the Ebola outbreak in Sierra Leone. Malar J.

[5] Eisele TP, Bennett A, Silumbe K, Finn TP, Chalwe V, Kamuliwo M (2016). Short-term impact of mass drug administration with dihydroartemisinin plus piperaquine on malaria in southern province Zambia: a cluster-randomized controlled trial. J Infect Dis.

[6] Tripura R, Peto TJ, Chea N, Chan D, Mukaka M, Sirithiranont P (2018). A controlled trial of mass drug administration to interrupt transmission of multidrug-resistant falciparum malaria in Cambodian villages. Clin Infect Dis.

[7] Landier Jordi, Kajeechiwa Ladda, Thwin May Myo, Parker Daniel M., Chaumeau Victor, Wiladphaingern Jacher, Imwong Mallika, Miotto Olivo, Patumrat Krittaya, Duanguppama Jureeporn, Cerqueira Dominique, Malleret Benoit, Rénia Laurent, Nosten Suphak, von Seidlein Lorenz, Ling Clare, Proux Stéphane, Corbel Vincent, Simpson Julie A., Dondorp Arjen M., White Nicholas J., Nosten François H. (2017). Safety and effectiveness of mass drug administration to accelerate elimination of artemisinin-resistant falciparum malaria: A pilot trial in four villages of Eastern Myanmar. Wellcome Open Research.

[8] Coldiron ME, Von Seidlein L, Grais RF (2017). Seasonal malaria chemoprevention: successes and missed opportunities. Malar J.

[9] Newby G, Hwang J, Koita K, Chen I, Greenwood B, von Seidlein L (2015). Review of mass drug administration for malaria and its operational challenges. Am J Trop Med Hyg.

[10] Wilson AL, Taskforce on behalf of the Ipt (2011). A systematic review and meta-analysis of the efficacy and safety of intermittent preventive treatment of malaria in children (IPTc). PLoS One.

[11] Aponte JJ, Schellenberg D, Egan A, Breckenridge A, Carneiro I, Critchley J (2009). Efficacy and safety of intermittent preventive treatment with sulfadoxine-pyrimethamine for malaria in African infants: a pooled analysis of six randomised, placebo-controlled trials. Lancet.

[12] Bell D, Wongsrichanalai C, Barnwell JW (2006). Ensuring quality and access for malaria diagnosis: how can it be achieved?. Nat Rev Microbiol Lond.

[13] Abeku TA, Kristan M, Jones C, Beard J, Mueller DH, Okia M (2008). Determinants of the accuracy of rapid diagnostic tests in malaria case management: evidence from low and moderate transmission settings in the East African highlands. Malar J.

[14] D’Acremont V, Lengeler C, Mshinda H, Mtasiwa D, Tanner M, Genton B (2009). Time to move from presumptive malaria treatment to laboratory-confirmed diagnosis and treatment in African children with fever. PLoS Med.

[15] Maltha J, Gillet P, Jacobs J (2013). Malaria rapid diagnostic tests in endemic settings. Clin Microbiol Infect.

[16] Lubell Y, Reyburn H, Mbakilwa H, Mwangi R, Chonya S, Whitty CJ (2008). The impact of response to the results of diagnostic tests for malaria: cost-benefit analysis. BMJ.

[17] Uzochukwu BS, Obikeze EN, Onwujekwe OE, Onoka CA, Griffiths UK (2009). Cost-effectiveness analysis of rapid diagnostic test, microscopy and syndromic approach in the diagnosis of malaria in Nigeria: implications for scaling-up deployment of ACT. Malar J.

[18] Ansah EK, Epokor M, Whitty CJM, Yeung S, Hansen KS (2013). Cost-effectiveness analysis of introducing RDTs for malaria diagnosis as compared to microscopy and presumptive diagnosis in central and peripheral public health facilities in Ghana. Am J Trop Med Hyg.

[19] Thiam S, Thior M, Faye B, Ndiop M, Diouf ML, Diouf MB (2011). Major reduction in anti-malarial drug consumption in Senegal after nation-wide introduction of malaria rapid diagnostic tests. PLoS One.

[20] Silumbe K, Yukich JO, Hamainza B, Bennett A, Earle D, Kamuliwo M (2015). Costs and cost-effectiveness of a large-scale mass testing and treatment intervention for malaria in Southern Province, Zambia. Malar J.

[21] Halliday KE, Okello G, Turner EL, Njagi K, Mcharo C, Kengo J, Allen E, Dubeck MM, Jukes MCH, Brooker SJ. Impact of intermittent screening and treatment for malaria among school children in Kenya: a cluster randomized trial [Internet]: The World Bank; 2014. p. 69. [cited 2019 Sep 29]. (Policy Research Working Papers). Available from: https://elibrary.worldbank.org/doi/abs/10.1596/1813-9450-6791. 10.1371/journal.pmed.1001594 PMC3904819 24492859

[22] Tiono AB, Ouédraogo A, Ogutu B, Diarra A, Coulibaly S, Gansané A (2013). A controlled, parallel, cluster-randomized trial of community-wide screening and treatment of asymptomatic carriers of Plasmodium falciparum in Burkina Faso. Malar J.

[23] Okella L, Slater H, Ghani A, Pemberton-Rossb P, Smith TA, Chitnis N, et al. Consensus modelling evidence to support the design of mass drug administration programmes. In: Malaria Policy Advisory Committee meeting. In; 2015. p. 16–8.

[24] Slater HC, Ross A, Ouédraogo AL, White LJ, Nguon C, Walker PGT, et al. Assessing the impact of next-generation rapid diagnostic tests on *Plasmodium falciparum* malaria elimination strategies [Internet]. Nature. 2015; [cited 2018 Mar 9]. Available from: https://www.nature.com/articles/nature16040. 10.1038/nature16040 26633771

[25] Brady OJ, Slater HC, Pemberton-Ross P, Wenger E, Maude RJ, Ghani AC (2017). Role of mass drug administration in elimination of Plasmodium falciparum malaria: a consensus modelling study. Lancet Glob Health.

[26] WHO | Cost-effectiveness of malaria diagnostic methods in sub-Saharan Africa in an era of combination therapy [Internet]. WHO. [cited 2018 Jul 2]. Available from: http://www.who.int/bulletin/volumes/86/2/07-042259/en/. 10.2471/BLT.07.042259 PMC2647374 18297164

[27] WHO | Meeting of the Evidence Review Group meeting on mass drug administration for malaria [Internet]. WHO. World Health Organization; [cited 2020 Mar 11]. Available from: http://www.who.int/malaria/meetings/2018/erg-mass-drug-administration/en/.

[28] Organization WH (2017). Mass drug administration for falciparum malaria: a practical field manual.

[29] Kaehler N, Adhikari B, Cheah PY, Day NPJ, Paris DH, Tanner M (2019). The promise, problems and pitfalls of mass drug administration for malaria elimination: a qualitative study with scientists and policymakers. Int Health.

[30] World Health Organization (2016). World malaria report 2015 [Internet].

[31] Walker PGT, Griffin JT, Ferguson NM, Ghani AC (2016). Estimating the most efficient allocation of interventions to achieve reductions in Plasmodium falciparum malaria burden and transmission in Africa: a modelling study. Lancet Glob Health.

[32] Okell LC, Griffin JT, Kleinschmidt I, Hollingsworth TD, Churcher TS, White MJ (2011). The potential contribution of mass treatment to the control of Plasmodium falciparum malaria. PLoS One.

[33] Gerardin J, Bever CA, Hamainza B, Miller JM, Eckhoff PA, Wenger EA (2016). Optimal population-level infection detection strategies for malaria control and elimination in a spatial model of malaria transmission. PLoS Comput Biol.

[34] Abba K, Deeks JJ, Olliaro PL, Naing C-M, Jackson SM, Takwoingi Y, et al. Rapid diagnostic tests for diagnosing uncomplicated *P. falciparum* malaria in endemic countries. In: Cochrane Database of Systematic Reviews [Internet]: Wiley; 2011. Available from: http://onlinelibrary.wiley.com/doi/10.1002/14651858.CD008122.pub2/abstract. 10.1002/14651858.CD008122.pub2 PMC6532563 21735422

[35] Moody A (2002). Rapid diagnostic tests for malaria parasites. Clin Microbiol Rev.

[36] Murray CK, Gasser RA, Magill AJ, Miller RS (2008). Update on rapid diagnostic testing for malaria. Clin Microbiol Rev.

[37] Valle D, Millar J, Amratia P (2016). Spatial heterogeneity can undermine the effectiveness of country-wide test and treat policy for malaria: a case study from Burkina Faso. Malar J.

[38] Drake TL, Lubell Y (2017). Malaria and economic evaluation methods: challenges and opportunities. Appl Health Econ Health Policy.

[39] Bhorat H, Kanbur R, Stanwix B (2017). Minimum wages in sub-Saharan Africa: a primer. World Bank Res Obs.

[40] Institut National de la Statistique et de la Démographie - INSD/Burkina Faso, ICF International (2012). Burkina Faso Enquête Démographique et de Santé et à Indicateurs Multiples (EDSBF-MICS IV) 2010 [Internet].

[41] Institut National de la Statistique - INS/Côte d'Ivoire, ICF International (2013). Côte d'Ivoire Enqêute Démographique et de Santé et à Indicateurs Multiples 2011–2012 [Internet].

[42] Ghana Statistical Service (2012). Ghana multiple Indicator cluster survey with an enhanced malaria module and biomarker 2011 [internet].

[43] Direction Nationale de la Statistique - DNS/Guinée, ORC Macro (2006). Guinée Enquête Démographique et de Santé 2005 [Internet].

[44] National Population Commission - NPC/Nigeria, ICF International (2014). Nigeria Demographic and Health Survey 2013 [Internet].

[45] Ministère de la Planification, du Développement et de l'Aménagement du Territoire (MPDAT), Ministère de la. Santé - MS/Togo, ICF International (2015). Togo Enquête Démographique et de Santè 2013–2014 [Internet].

[46] Hamer DH, Ndhlovu M, Zurovac D, Fox M, Yeboah-Antwi K, Chanda P (2007). Improved diagnostic testing and malaria treatment practices in Zambia. JAMA..

[47] Wongsrichanalai C, Barcus MJ, Muth S, Sutamihardja A, Wernsdorfer WH (2007). A review of malaria diagnostic tools: microscopy and rapid diagnostic test (RDT). Am J Trop Med Hyg.

[48] R Core Team. R: a language and environment for statistical computing [internet]. Vienna, Austria: R Foundation for Statistical Computing; 2017. Available from: https://www.R-project.org/.

[49] brms: An R Package for Bayesian Multilevel Models Using Stan | Bürkner | Journal of Statistical Software. [cited 2018 Sep 26]; Available from: https://www.jstatsoft.org/article/view/v080i01.

[50] Gelman A (2006). Prior distributions for variance parameters in hierarchical models (comment on article by Browne and Draper). Bayesian Anal.

[51] Bürkner P-C (2018). Advanced Bayesian multilevel modeling with the R package brms. R J.

[52] Gelman A, Rubin DB (1992). Inference from iterative simulation using multiple sequences. Stat Sci.

[53] Chang W, Cheng J, Allaire JJ, Xie Y, McPherson J (2017). shiny: web application framework for R [Internet].

[54] Smith CM, Hayward AC (2016). DotMapper: an open source tool for creating interactive disease point maps. BMC Infect Dis.

[55] Jombart T, Aanensen DM, Baguelin M, Birrell P, Cauchemez S, Camacho A (2014). OutbreakTools: a new platform for disease outbreak analysis using the R software. Epidemics..

[56] Moraga P (2017). SpatialEpiApp: a Shiny web application for the analysis of spatial and spatio-temporal disease data. Spat Spatio-Temporal Epidemiol.

[57] Vilalta C, Arruda AG, Tousignant SJP, Valdes-Donoso P, Muellner P, Muellner U (2017). A review of quantitative tools used to assess the epidemiology of porcine reproductive and respiratory syndrome in U.S. swine farms using Dr. Morrison’s swine health monitoring program data. Front Vet Sci.

[58] Cheng J, Karambelkar B, Xie Y (2017). leaflet: create interactive web maps with the JavaScript “Leaflet” library [Internet].

[59] Tiono AB, Guelbeogo MW, Sagnon NF, Nébié I, Sirima SB, Mukhopadhyay A (2013). Dynamics of malaria transmission and susceptibility to clinical malaria episodes following treatment of Plasmodium falciparum asymptomatic carriers: results of a cluster-randomized study of community-wide screening and treatment, and a parallel entomology study. BMC Infect Dis.

[60] English M, Reyburn H, Goodman C, Snow RW (2009). Abandoning presumptive antimalarial treatment for febrile children aged less than five years—a case of running before we can walk?. PLoS Med.

[61] Reyburn H, Mbakilwa H, Mwangi R, Mwerinde O, Olomi R, Drakeley C (2007). Rapid diagnostic tests compared with malaria microscopy for guiding outpatient treatment of febrile illness in Tanzania: randomised trial. BMJ.

[62] Kilian AH, Metzger WG, Mutschelknauss EJ, Kabagambe G, Langi P, Korte R (2000). Reliability of malaria microscopy in epidemiological studies: results of quality control. Trop Med Int Health TM IH.

[63] Mappin B, Cameron E, Dalrymple U, Weiss DJ, Bisanzio D, Bhatt S (2015). Standardizing Plasmodium falciparum infection prevalence measured via microscopy versus rapid diagnostic test. Malar J.

[64] Okell LC, Bousema T, Griffin JT, Ouédraogo AL, Ghani AC, Drakeley CJ (2012). Factors determining the occurrence of submicroscopic malaria infections and their relevance for control. Nat Commun.

[65] Gerardin J, Ouédraogo AL, McCarthy KA, Eckhoff PA, Wenger EA (2015). Characterization of the infectious reservoir of malaria with an agent-based model calibrated to age-stratified parasite densities and infectiousness. Malar J.

[66] Orish VN, Ansong JY, Onyeabor OS, Sanyaolu AO, Oyibo WA, Iriemenam NC (2016). Overdiagnosis and overtreatment of malaria in children in a secondary healthcare centre in Sekondi-Takoradi, Ghana. Trop Doct.

[67] Nyangena O (2018). Malaria overtreatment still very common in Kenya despite negative malaria tests. Am J Clin Pathol.

[68] Salomão CA, Sacarlal J, Chilundo B, Gudo ES (2015). Prescription practices for malaria in Mozambique: poor adherence to the national protocols for malaria treatment in 22 public health facilities. Malar J.

[69] Rabinovich RN, Drakeley C, Djimde AA, Hall BF, Hay SI, Hemingway J (2017). malERA: an updated research agenda for malaria elimination and eradication. PLoS Med.

[70] Lubell Y, Hopkins H, Whitty CJ, Staedke SG, Mills A (2008). An interactive model for the assessment of the economic costs and benefits of different rapid diagnostic tests for malaria. Malar J.

[71] Kelly GC, Tanner M, Vallely A, Clements A (2012). Malaria elimination: moving forward with spatial decision support systems. Trends Parasitol.

[72] Kelly GC, Seng CM, Donald W, Taleo G, Nausien J, Batarii W (2011). A spatial decision support system for guiding focal indoor residual spraying interventions in a malaria elimination zone. Geospat Health.

[73] Wangdi K, Banwell C, Gatton ML, Kelly GC, Namgay R, Clements AC (2016). Development and evaluation of a spatial decision support system for malaria elimination in Bhutan. Malar J.

[74] Bainbridge I (2014). PRACTITIONER’S PERSPECTIVE: how can ecologists make conservation policy more evidence based? Ideas and examples from a devolved perspective. J Appl Ecol.

[75] Ghana Statistical Service - GSS, Ghana Health Service - GHS, ICF International (2015). Ghana Demographic and Health Survey 2014 [Internet].

[76] Valle D, Toh KB, Millar J. Rapid prototyping of decision-support tools for conservation. Conserv Biol. 2019; [cited 2019 Sep 25]; Available from: https://conbio.onlinelibrary.wiley.com/doi/10.1111/cobi.13305. 10.1111/cobi.13305 30829420

