# Supplementary material for: To screen or not to screen: an interactive framework for comparing costs of mass malaria treatment interventions
Source: BMC Med. 2020 Jun 19;18:149. doi: 10.1186/s12916-020-01609-7 (PMC7304173; doi:10.1186/s12916-020-01609-7)
Supplement: Supplementary file 1 — Additional file 1. [file 12916_2020_1609_MOESM1_ESM.pdf]

Supplemental Material

Model Specification and full posterior distribution

$$
\begin{aligned}
M_{ijk} &\sim \text{Bernoulli}(p_{ijk}) \\
g(p_{ijk}) &= \alpha_{jk} + \beta_{0,k} + \beta_{1,k} * Age_{ijk} + \beta_{2,k} * Urban_{jk} + \beta_3 * Age_{ijk} + \beta_4 * Urban_{jk} \\
p(\beta_{l,k}) &\sim 1, \text{for}(l = 0, 1, 2) \\
p(\beta_l) &\sim 1, \text{for}(l = 3, 4) \\
\alpha_{jk} &\sim N(0, \sigma^2_{\alpha_k}) \\
\sigma_{\alpha_k} &\sim \text{half-}t(3, 0, 10)
\end{aligned}
$$

Here we present the full model specification and complete posterior distribution. The malaria status M_{ijt} (1 if infected, 0 if not) of individual i from cluster j in region k follows a Bernoulli distribution. Applying a logit-link function $g(p_{ijk}) = log(p/1-p)$, we estimate p_{ijk} using a mixed-effect model where α_{jk} are intercepts for each cluster j in region k, β are the is the population-level effects (sometimes referred to as "fixed" effects) and β_k are group-level effects (sometimes called "random effects"). Note that the model contains random intercepts and random slopes at the region k level. The priors were selected to be non-informative (Gelman and others 2006), and are the default prior choices in the `brms` package (Bürkner and others 2017).

Based on these parameters, the full posterior distribution of our model is:

$$
P(\sigma, \alpha, \beta, |...) \propto (\prod_{i,j,k} \text{Bernoulli}(M_{ijk}|p_{ijk}))(\prod_{j,k} N(\alpha_{jk}|0, \sigma^2_{\alpha_k}))(\prod_{k} \text{half-}t(\sigma_{\alpha_k}|3, 0, 10))
$$

Model outputs from Burkina Faso.

Here we provide some diagnostic information on for our prevalence, sensitivity, and specificity models, for the Burkina Faso data. As we explicitly discuss in the main article, one of the primary goals of this work is to demonstrate the usefulness of extending a relatively standard statistical model into an interactive tool for decision support. As such, our models rely on widely used open source data, and both tools and models are intentionally designed to be relatively simple.

Prevalence Model

Gelman-Rubin convergence values ($\hat{R}$) range from 0.999 to 1.003.

Trace plots

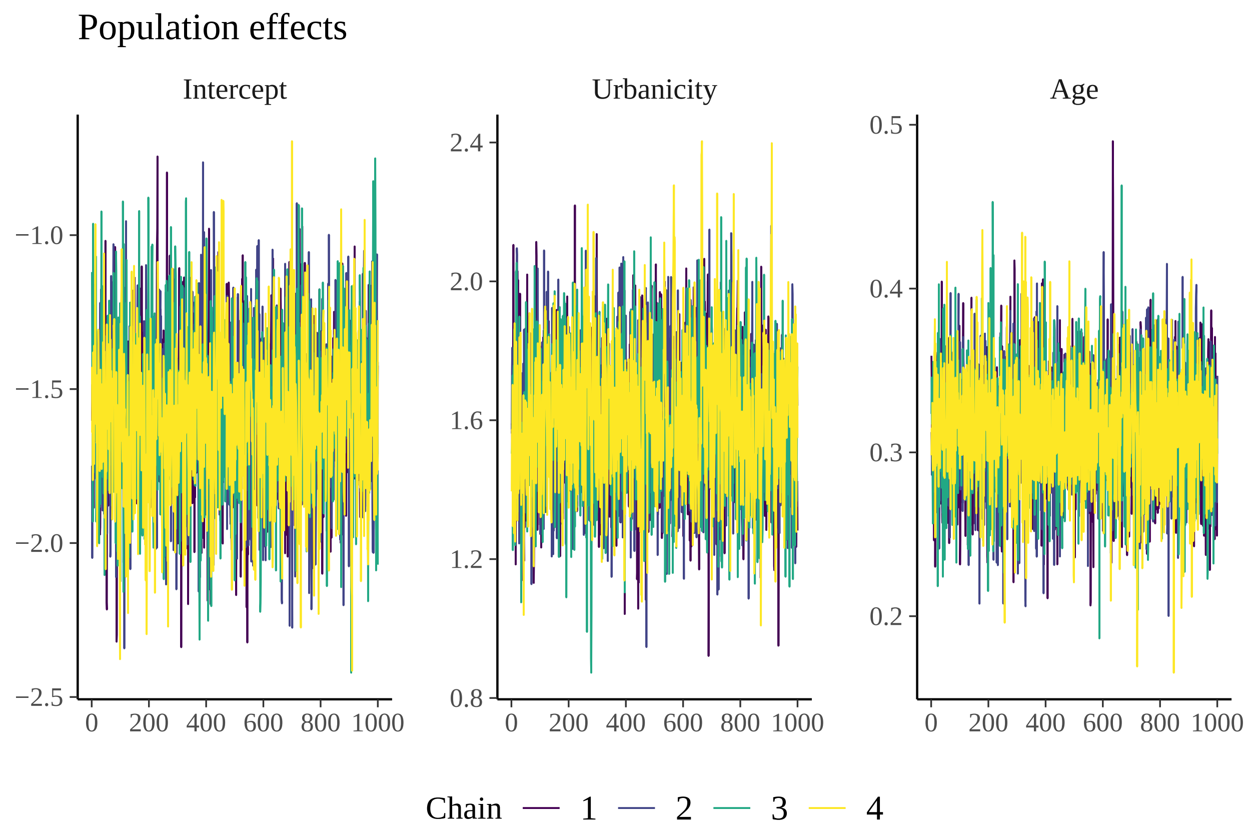
Population effects
Intercept
Urbanicity
Age
−1.0
−1.5
−2.0
−2.5
2.4
2.0
1.6
1.2
0.8
0.5
0.4
0.3
0.2
0
200
400
600
800
1000
Chain
1
2
3
4

Urban/Rural Regional Effects

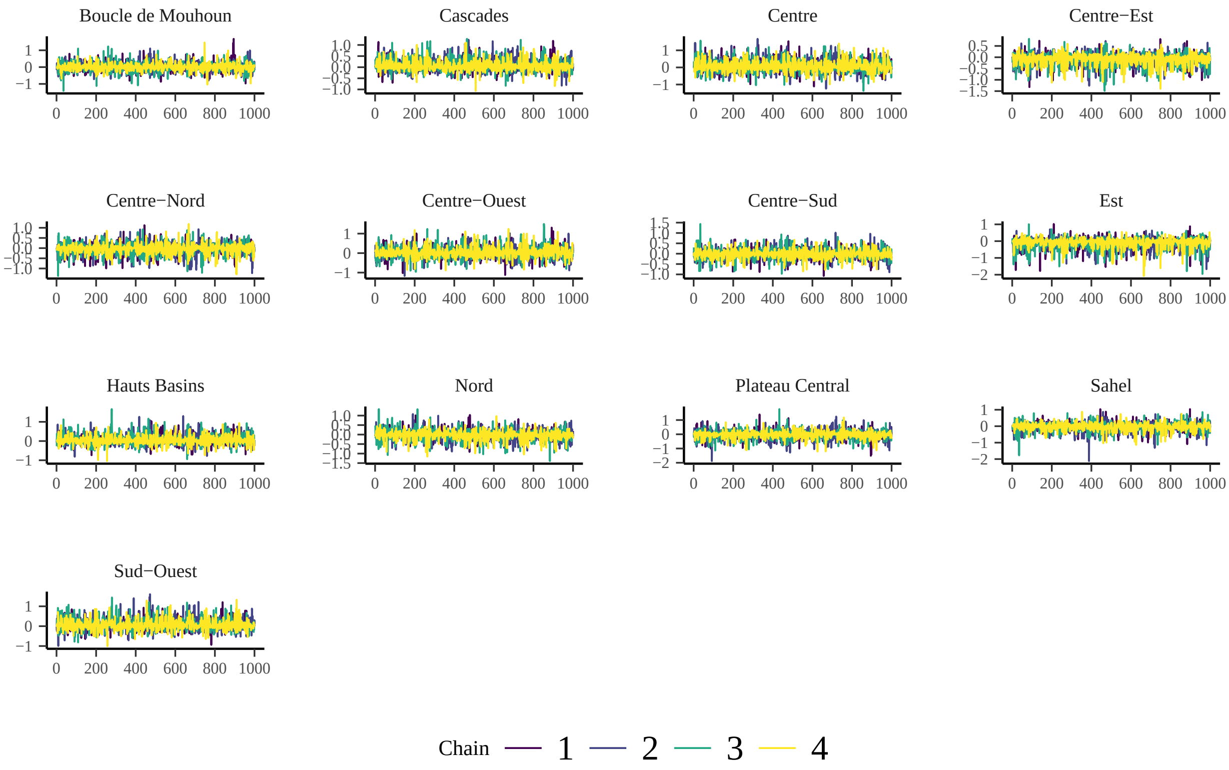

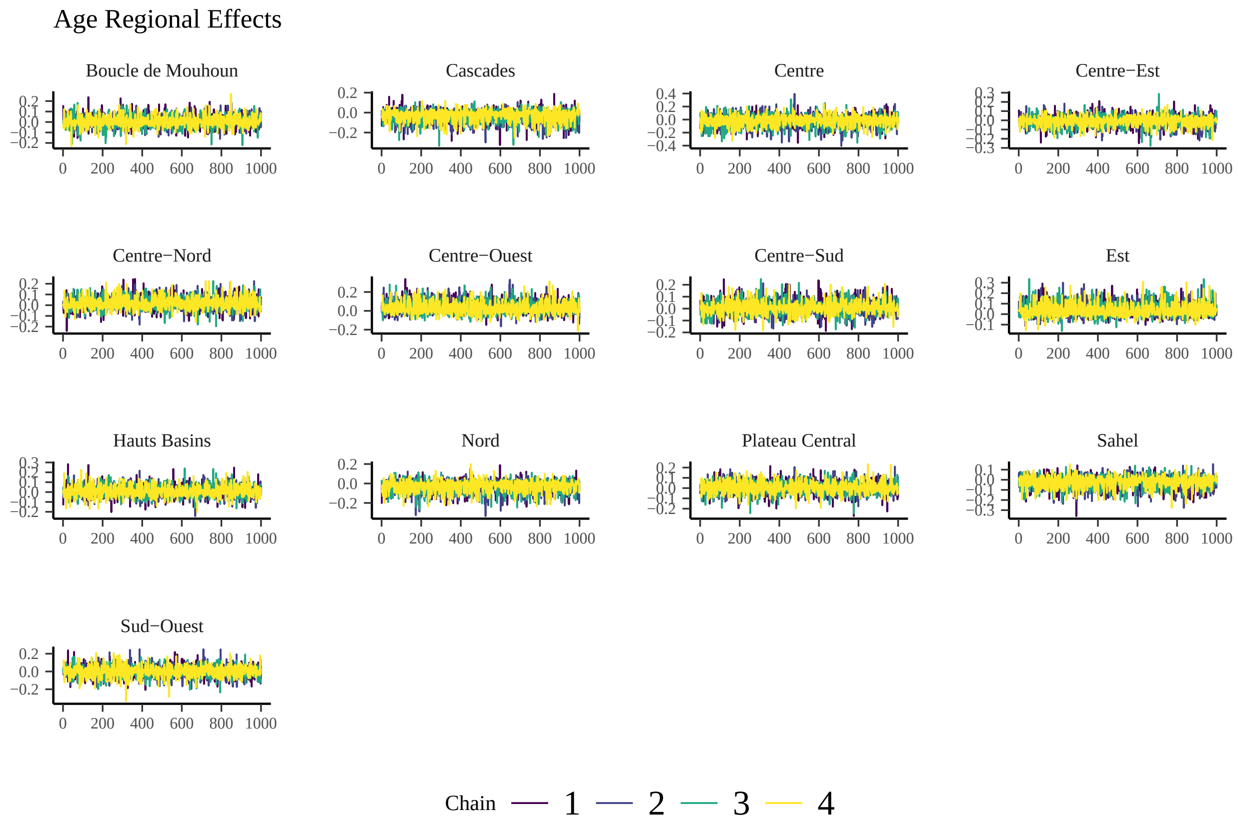

Sensitivity Model

Gelman-Rubin convergence values ($\hat{R}$) range from 0.999 to 1.005.

Trace plots

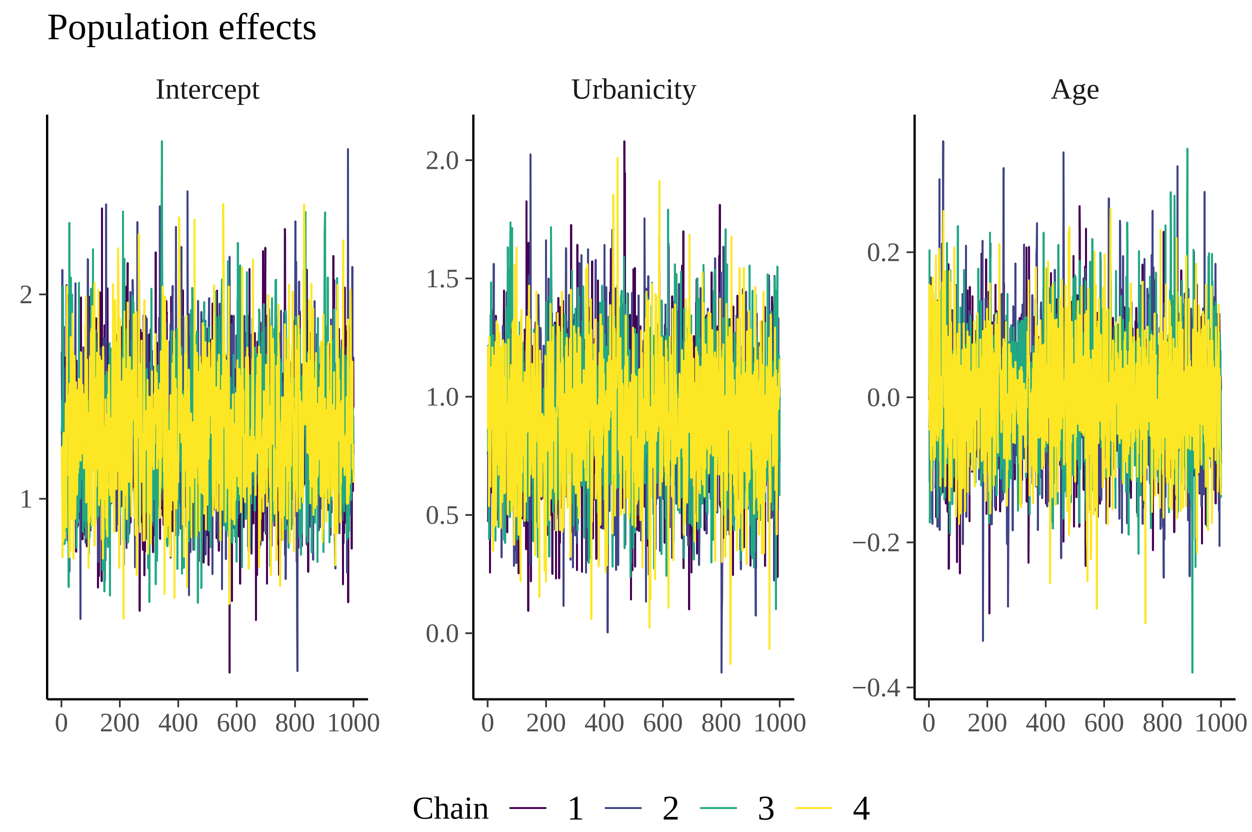
Population effects
Intercept
Urbanicity
Age
2
1
2.0
1.5
1.0
0.5
0.0
0.2
0.0
−0.2
−0.4
0
200
400
600
800
1000
Chain
1
2
3
4

Urban/Rural Regional Effects

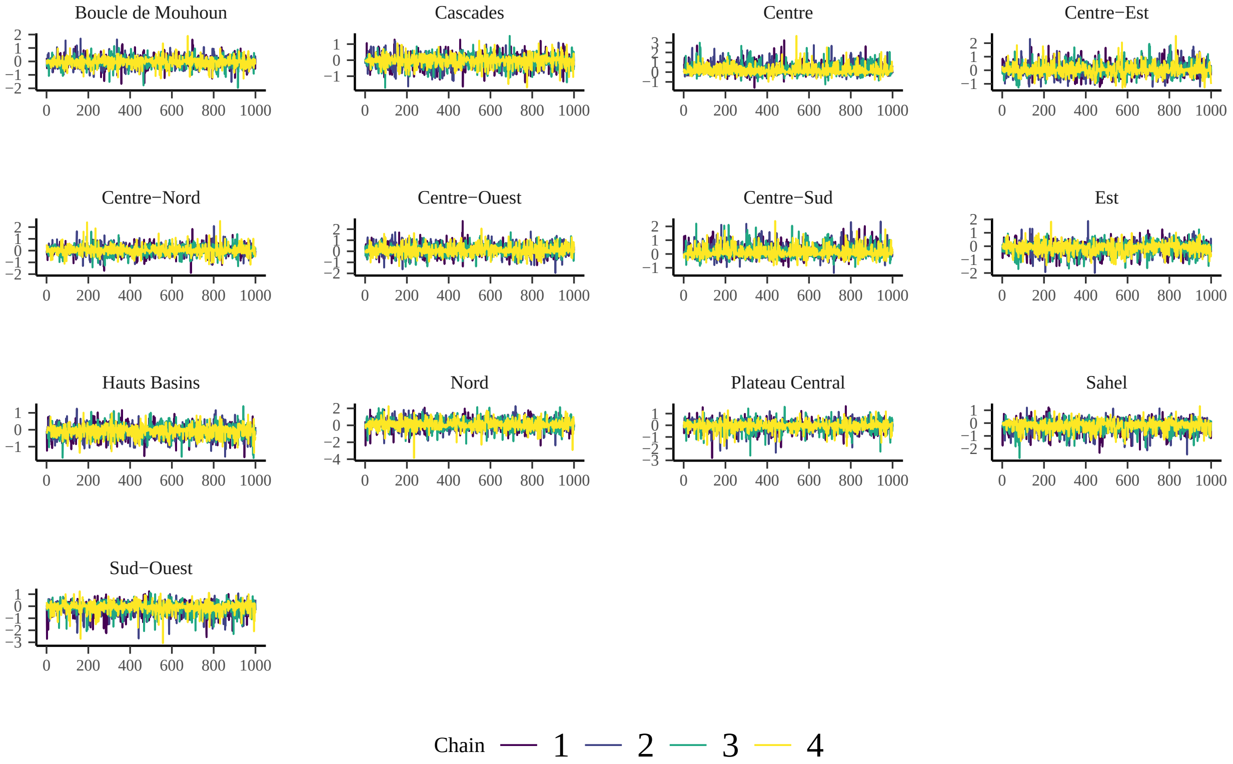

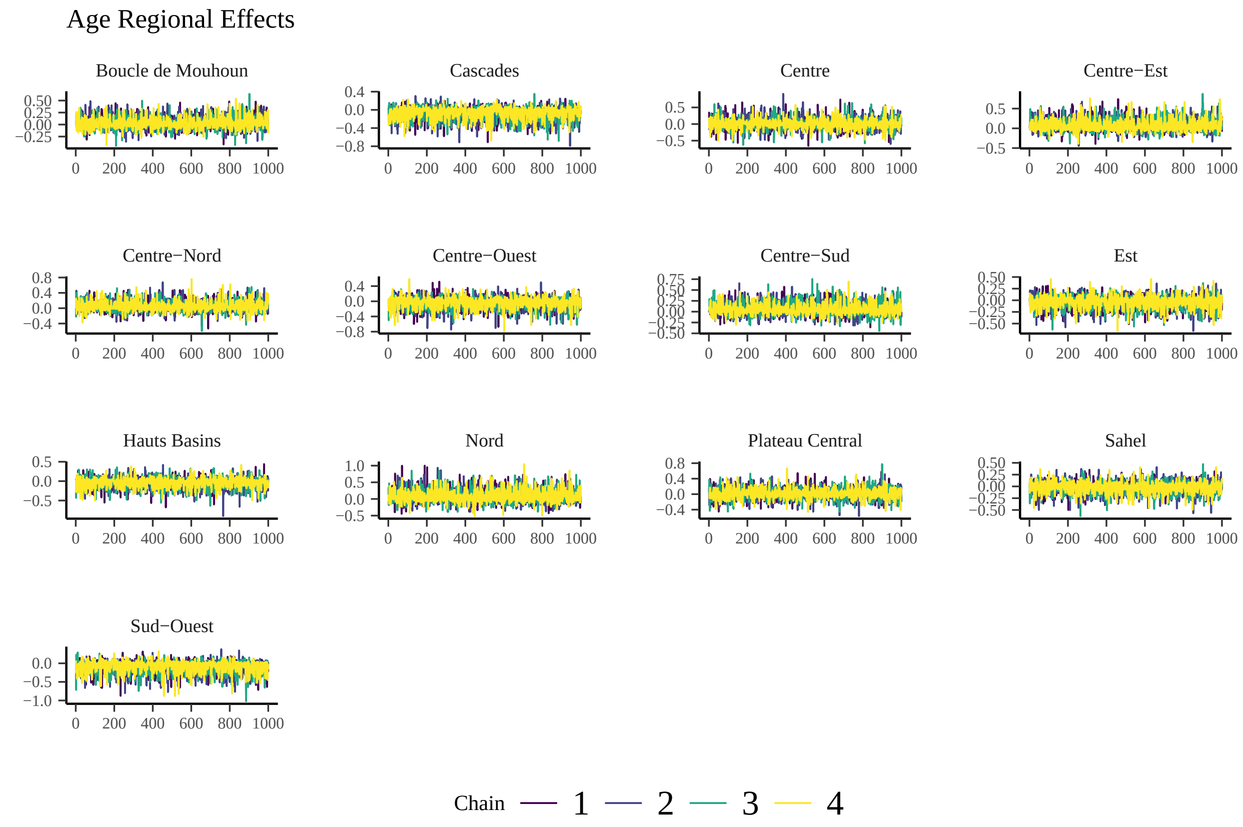

Specificity Model

Gelman-Rubin convergence values ($\hat{R}$) range from 0.999 to 1.005.

Trace plots

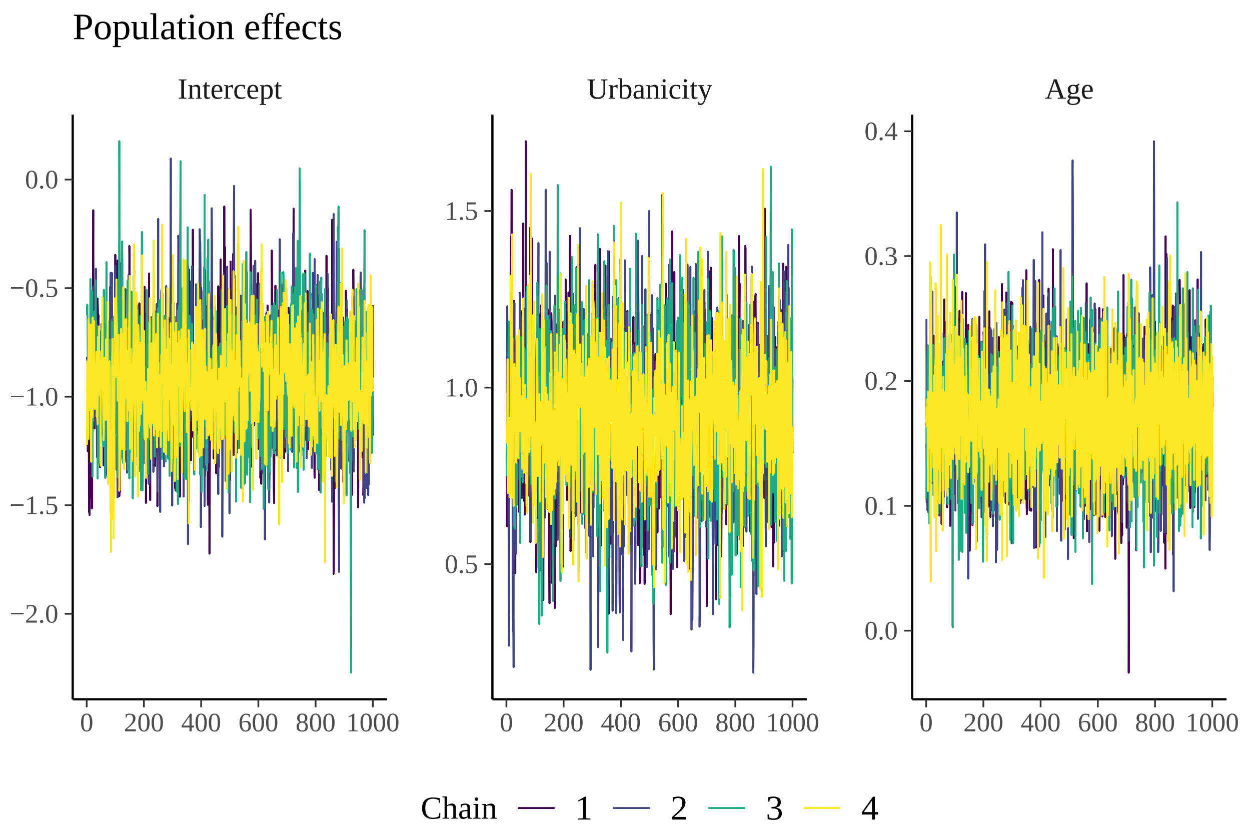
Population effects
Intercept
Urbanicity
Age
0.0
−0.5
−1.0
−1.5
−2.0
1.5
1.0
0.5
0.4
0.3
0.2
0.1
0.0
0
200
400
600
800
1000
Chain
1
2
3
4

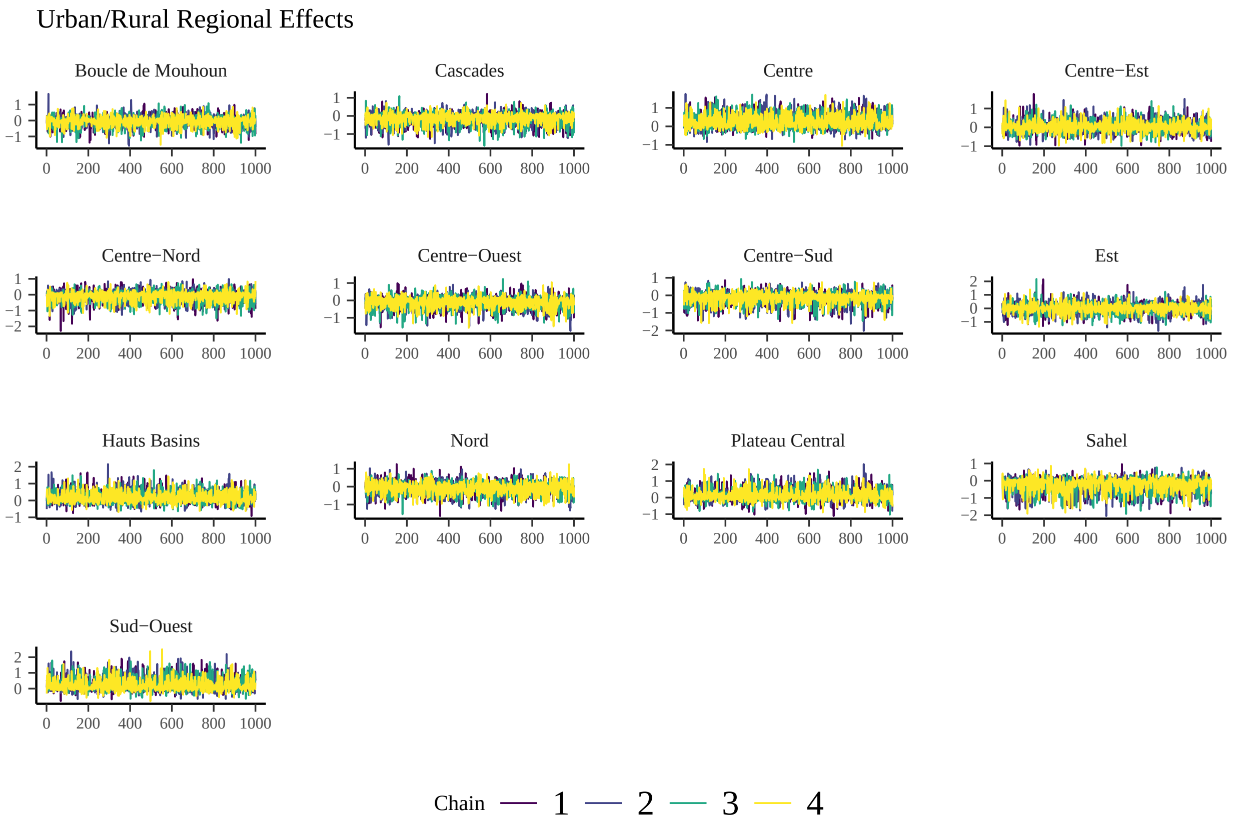
Urban/Rural Regional Effects
Boucle de Mouhoun
Cascades
Centre
Centre−Est
Centre−Nord
Centre−Ouest
Centre−Sud
Est
Hauts Basins
Nord
Plateau Central
Sahel
Sud−Ouest
0 200 400 600 800 1000
Chain 1 2 3 4

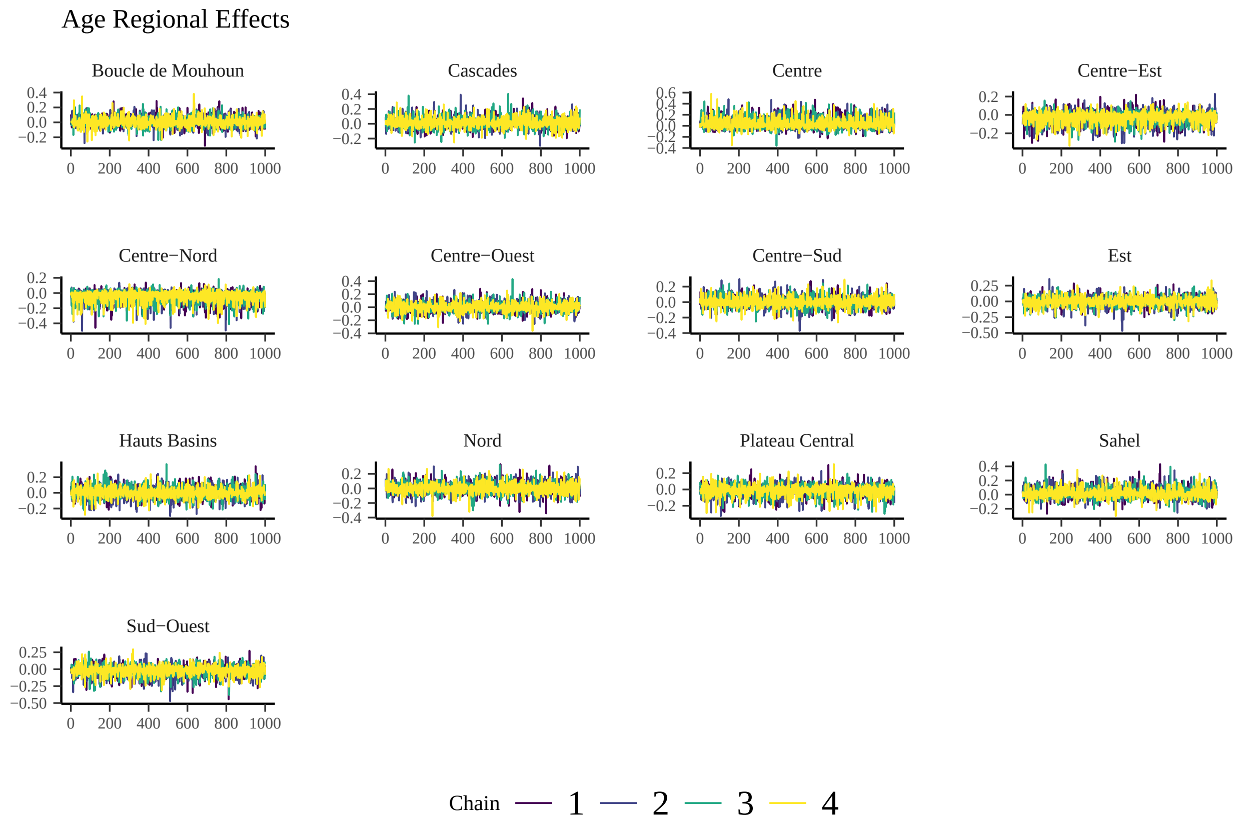

References

Bürkner, Paul-Christian, and others. 2017. "Brms: An R Package for Bayesian Multilevel Models Using Stan." *Journal of Statistical Software* 80 (1): 1–28.

Gelman, Andrew, and others. 2006. "Prior Distributions for Variance Parameters in Hierarchical Models (Comment on Article by Browne and Draper)." *Bayesian Analysis* 1 (3): 515–34.
